# Impact of Mg substitution in LaMnO_3_ manganites on their structural integrity and magnetic behavior†

**DOI:** 10.1039/d4ra08238a

**Published:** 2025-03-19

**Authors:** Parvathy Namboothiri, Vishnumaya K. J., Phuong V. Pham, K. K. Supin, M. Vasundhara

**Affiliations:** a Polymers and Functional Materials Department, CSIR-Indian Institute of Chemical Technology Hyderabad-500007 India mvas@iict.res.in vasu.mutta@gmail.com; b Academy of Scientific and Innovative Research (AcSIR) Ghaziabad-201002 India; c Department of Physics, National Sun Yat-sen University Kaohsiung 80424 Taiwan

## Abstract

In this study, we investigated the impact of Mg substitution at the La site in LaMnO_3_ on its structural, chemical, and magnetic properties. To explore the effects of Mg doping, we synthesized a series of compositions, La_1−*x*_Mg_*x*_MnO_3_ (*x* = 0.05, 0.1, 0.15, 0.2, 0.3, and 0.33), using a conventional solid-state method and systematically investigated their structural and magnetic properties. X-Ray diffraction patterns confirmed the formation of a pure rhombohedral crystal structure for the samples up to *x* = 0.15. However, for compositions with *x* > 0.15, the emergence of a secondary phase, MgMn_2_O_4_ with a spinel structure, was observed alongside the primary phase. Furthermore, it was observed that the secondary phase systematically increases with increasing Mg concentration. The temperature variations in magnetic studies, measured under zero-field-cooled (ZFC) and field-cooled (FC) conditions, confirmed that the transition temperatures for all the samples were found to be below 200 K, and these temperatures were observed to decrease with increasing Mg content. Negative magnetization was observed in the low-temperature ZFC curves for the samples with *x* > 0.2, indicating a difference in temperature-dependent magnetization due to the presence of two different structural phases. This effect became more prominent with the increase in the secondary phase, MgMn_2_O_4_. The magnetic studies revealed the co-existence of ferromagnetic and ferrimagnetic ordering in La_1−*x*_Mg_*x*_MnO_3_ for *x* > 0.15 samples, while ferromagnetic ordering was retained for the samples with *x* < 0.15, which was further corroborated by the isothermal hysteresis results.

## Abstract 2

In this study, we investigated the impact of Mg substitution at the La site in LaMnO_3_ on its structural, chemical, and magnetic properties.

## Graphical abstract

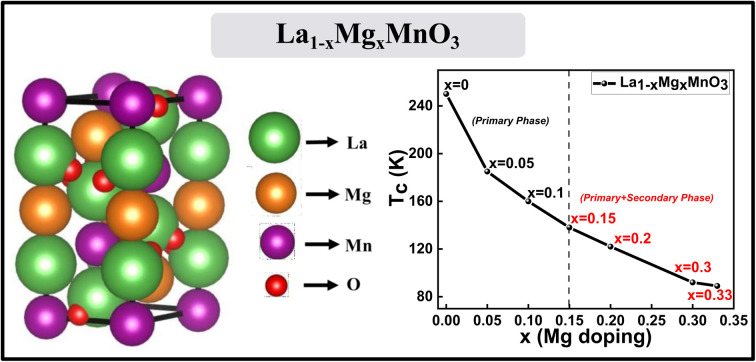

## 1. Introduction

Perovskite compounds with the generic formula ABO_3_, where A is an RE element and B is a transition metal element, exhibit a wide range of interesting physical properties, making them valuable materials for several technological applications. These properties include ferroelectric, dielectric, pyroelectric, and piezoelectric behaviour.^1–4^ They have also found applications in solar cells, light-emitting diodes, photodetectors, ferroelectric devices, piezoelectric devices, catalysts, gas sensors, magnetic and spintronic devices, supercapacitors, water splitting and O_2_ evolution, and thermoelectric devices.^5–15^ LaMnO_3_ (LMO) is a perovskite manganite with a rhombohedral crystal structure, exhibiting complex magnetic behaviours, multiferroic properties, and potential applications in catalysis and high-temperature environments, making it a subject of extensive study in materials science. LMO is known to be a layer-type (A-type) antiferromagnet with a Néel temperature around 170 K, indicating that the antiferromagnetic order sets in at relatively low temperatures.^16,17^ In the 1950s, pioneering studies by Jonker and Van Santen^18^ revealed that varying the proportion of Mn ions through the introduction of bivalent alkaline earth metals (*e.g.*, Mg, Ca, Sr, and Ba) into LMO resulted in drastic changes in their magnetic and electrical properties, which eventually led to several technologically important properties. The inclusion of bivalent alkali elements at the La site in LMO through substitution leads to the formation of Mn^4+^ ions in LMO, resulting in a mixed valence of Mn^3+^ and Mn^4+^ ions in order to maintain the overall charge neutrality of the compound. Consequently, the term “mixed-valent manganites” is employed to describe substances that incorporate Mn in various ionic states.^19^ The physical properties of LMO, including structural alterations and the Mn^4+^/Mn^3+^ ratio, are influenced by the partial replacement of La ions with other bivalent alkaline earth metals. This substitution gives rise to phenomena, such as charge and orbital ordering, governed by the interaction between electrons in the e_g_ and t_2g_ levels.^20^

La_1−*x*_A_*x*_MnO_3_ is as one of the extensively researched families within the realm of manganites, while the physical characteristics of Nd and Pr-based manganites differ due to the weak interaction arising from their smaller ionic radii.^21–26^ Numerous theories have been postulated to elucidate the underlying physics of manganites, including the double-exchange (DE) interaction, Jahn–Teller effect, and phase separation. In the hole-doped manganites,^27^ the co-existence of Mn^3+^ and Mn^4+^ oxidation states leads to ferromagnetism and conduction. This behaviour is commonly explained by the DE mechanism,^28^ wherein the magnetic coupling between Mn^3+^ and Mn^4+^ ions stems from the motion of an e_g_ electron between the two partially filled d-orbitals with strong on-site Hund's coupling. The magnetic properties are intricately linked to the strength of the DE interaction between Mn^3+^ and Mn^4+^ through oxygen atoms, making the Mn-site doping crucial for modifying this interaction strength. Notably, an Mn^4+^ content of 12% induces an orthorhombic configuration with antiferromagnetic ordering, whereas higher concentrations of Mn^4+^ result in a rhombohedral or cubic structure, showcasing ferromagnetism.^29,30^

A substitution in LMO with bivalent alkaline earth metals has indeed been a subject of significant research interest in recent years. In the specific case of LMO, alkaline earth metals like Ca, Sr, Pb and Ba are commonly used as dopants.^31–34^ This substitution has shown profound effects on the physical and chemical properties of the material. While Mg substitution in LMO has been previously investigated, the impact of varying doping rates on the structural and magnetic properties is of significant interest. Different synthesis techniques and calcination rates can lead to the formation of secondary phases with distinct structural and magnetic characteristics.^35^ The formation of a secondary phase has also been observed, prompting an exploration of the underlying chemical mechanisms responsible for its evolution. As suggested by Zhao *et al.*, the suppression of the metal-insulator transition with Mg substitution, unlike similar systems such as La_1−*x*_Sr_*x*_MnO_3_ or La_1−*x*_Ca_*x*_MnO_3_, and ferromagnetic ordering temperature peaks around *x* = 0.1 decreases with a higher Mg doping.^35^ Therefore, in this study, we investigated a systematic substitution of Mg in LMO, *i.e.*, La_1−*x*_Mg_*x*_MnO_3_ (where *x* = 0.05, 0.1, 0.15, 0.2, 0.3, and 0.33) compositions using conventional solid-state methods. The primary objective is to examine the impact of the Mg dopant at the La-site on the structural stability of these manganites. Further, a systematic investigation has been conducted to explore the repercussions of these structural variations on the morphological, compositional, and magnetic properties.

## 2. Experimental work

The synthesis of La_1−*x*_Mg_*x*_MnO_3_ with *x* = 0.05, 0.1, 0.15, 0.2, 0.3, and 0.33 (LMMO) was done using a conventional solid-state method. The compounds were prepared by taking La_2_O_3_ (Alfa Aesar, 99%), MgCO_3_ (Sigma Aldrich, 99.9%), and MnCO_3_ (Sigma Aldrich, 99.9%) as the raw materials. The reagents were stoichiometrically weighed, homogeneously mixed and dried and were calcined at different temperatures, *i.e.*, 900 °C, 1000 °C, and 1100 °C, each for 12 hours to obtain the phase pure perovskite structures. The powders, thus obtained, were pressed into circular pellets and finally sintered at 1250 °C for 12 hours in the air. It is important to note that after each consecutive calcination step, the compounds were thoroughly ground to ensure a uniform and thorough mixing of the particles. This grinding process is crucial for achieving homogeneity in the material, as it helps to distribute the heat-treated particles evenly. The uniformity in particle size and distribution can significantly impact the final properties and performance of the compounds, ensuring consistent results in the subsequent processing or applications. The X-ray diffraction data were obtained using a Bruker D8 Advance DaVinci diffractometer in the Bragg–Brentano (reflection) geometry. The instrument was equipped with a Cu K_α_ X-ray source (wavelength of 1.5418 Å) and a LYNXEYE detector, and the measurements were conducted at room temperature. The powder sample was placed in the sample holder and gently compressed with a glass slide to ensure a flat sample surface suitable for X-ray diffraction measurements in reflection geometry. X-rays were generated at 40 kV and 30 mA, and the measurements were carried out on a finely ground powder sample with a step size of 0.02 and covering the 2-theta range from 2.00° to 80.00°. The obtained diffractograms were processed using Bruker DIFFRAC.EVA software. The surface morphology and elemental analysis were conducted using the Field Emission Scanning Electron Microscopy/Energy Dispersive X-Ray Spectroscopy techniques, employing the Hitachi-S520 instrument from Japan with the Oxford link ISISSEM Model. Magnetic properties were studied using a vibrating sample magnetometer attached to physical property measurement equipment (Quantum Design Inc., USA).

## 3. Results and discussion

### 3.1 X-ray diffraction (XRD)

**Fig. 1:**
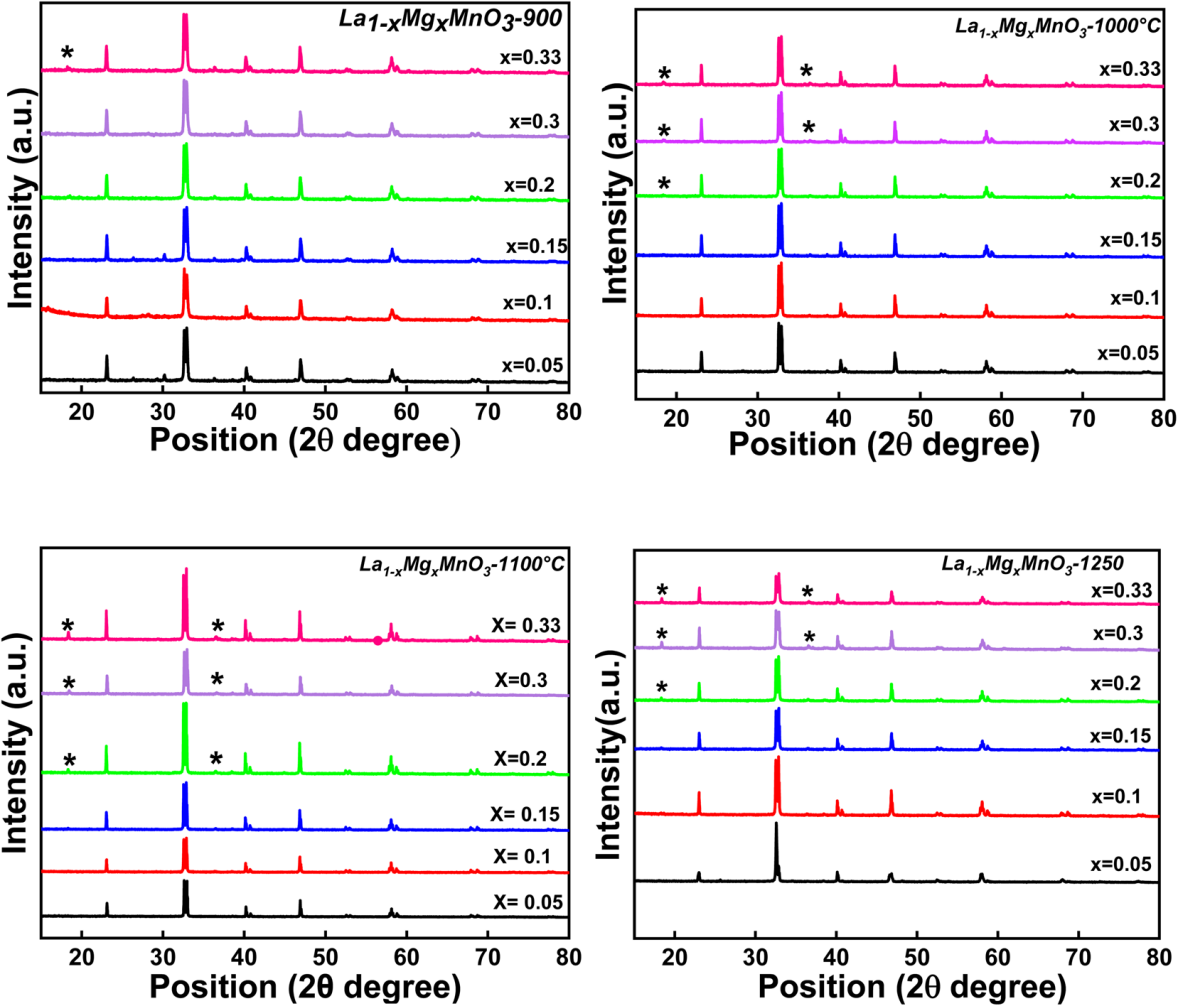
XRD patterns of LMMO_3_ calcined at 900 °C, 1000 °C, 1100 °C and 1250 °C. A regular increase in the intensity of the secondary phase MgMn_2_O_4_ is observed.

**Fig. 2:**
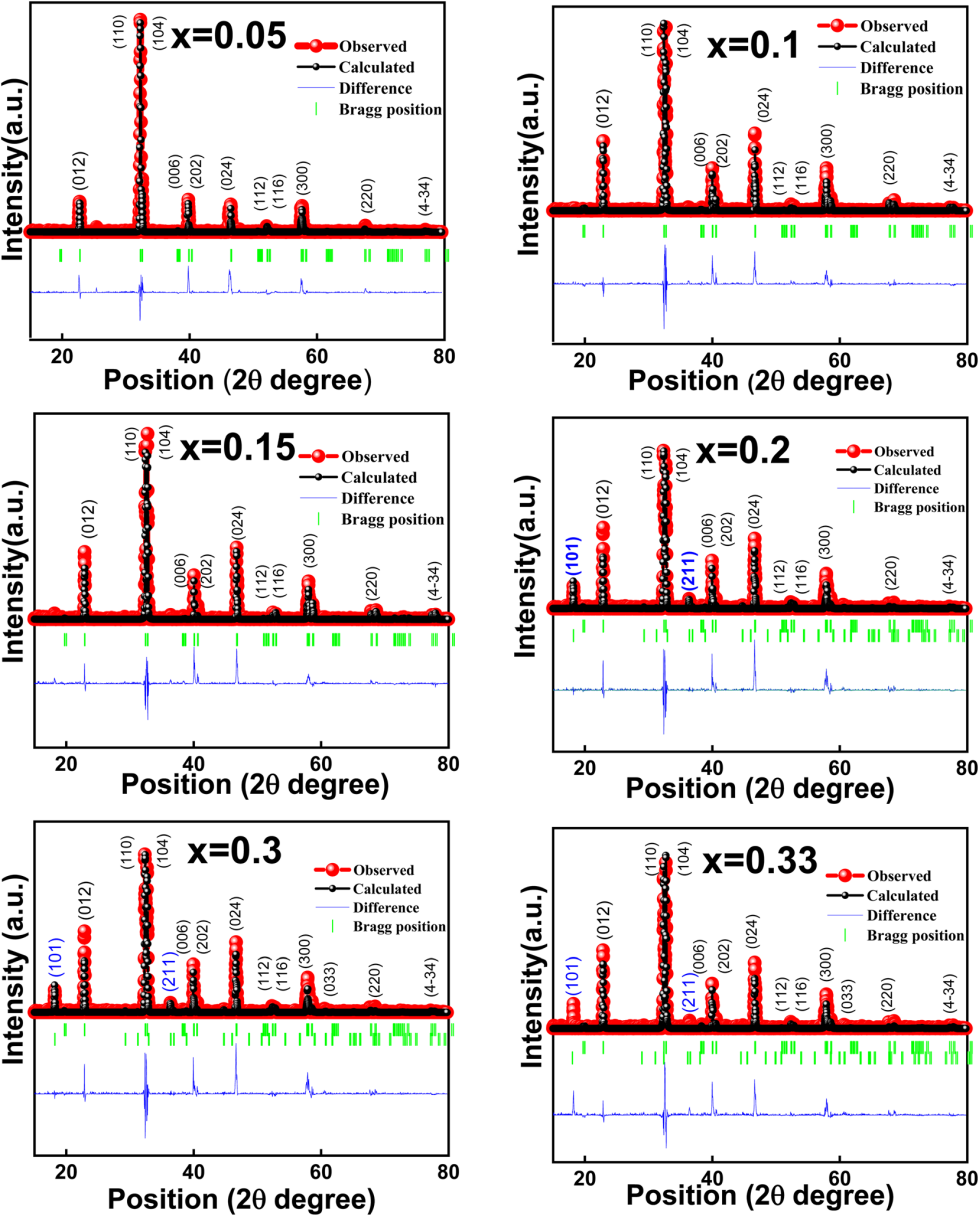
XRD patterns of LMMO sintered at 1250 °C after Rietveld refinement. Markings in blue represent the secondary phase.

LMO has been reported to crystallize into a rhombohedral perovskite structure.^36^ XRD analysis of all the studied LMMO compounds was performed systematically following each successive calcination process to investigate the development of crystal structures, as illustrated in Fig. 1. It is noticed that a perovskite structure is formed for all the compounds calcined at 900 °C; however, a secondary phase (indicated by an asterisk *) is evolved for higher Mg content samples. The same secondary phase is found to evolve with the increase in calcination temperatures, *i.e.*, at 1000 °C and 1100 °C. Thus, it is observed that upon an increase in the Mg-content and calcination temperatures, the secondary phase starts evolving prominently. Further, it is also observed that the intensity of the secondary peaks increases with an increase in the doping concentration of Mg and calcination temperature. The XRD patterns confirm a rhombohedral crystal structure with the *R*3̄ space group in all the LMMO compounds as per the Inorganic Crystal Structure Database (ICSD reference number: 98-010-4033). Finally, the XRD patterns recorded on the sintered samples indicated that the samples with *x* ≤ 0.15 compositions crystallised into a phase pure rhombohedral lattice with the space group *R*3̄ without any secondary peaks. However, the evolution of a secondary phase, which is identified as MgMn_2_O_4_ in a tetragonal lattice with the space group *I*4_1_/*amd*, results in extra peaks at 18.3° and 36.5° for compounds with *x* > 0.15 compositions. As the Mg doping concentration and calcination temperature increase, the percentage phase fraction of the secondary phase also rises consistently. In order to gain a deeper insight into the crystal structure, the XRD patterns of all the sintered samples were fitted using FullProf Software, as shown in Fig. 2. The experimental XRD patterns are in good agreement with the earlier observed patterns.^37^ Even though the formation of MgMn_2_O_4_ depends on the amount of doping, some reports show that the preparation conditions, such as manual grinding, sometimes do not ensure perfect homogeneity and produce the secondary phase of MgMn_2_O_4_. It shows that, with an increase in Mg content, the percentage of the MgMn_2_O_4_ secondary phase also increases. Contradicting the present work, J. H. Zhao *et al.* reported Mg-substituted compounds with compositions (0.05 < *x* < 0.4 and 0.45 < *x* < 0.6) as a single phase without any emerging secondary phases.^38,39^ Blasco *et al.* also reported a single phase for Mg-substitution up to *x* = 0.5.^40^ It is to be understood here that the sample preparation conditions described by J. H. Zhao *et al.* and Blasco *et al.* are ball milling techniques, which are highly efficient for preventing the formation of secondary phases. In addition, few researchers have reported the secondary phase, which is manganese oxides with the substitution of Mg, unlike the phase separation observed in the present work.^41^

However, in the present study, the synthesis process, which includes calcination and sintering, was conducted in a muffle furnace under ambient conditions, using traditional manual grinding with a mortar and pestle. Further, the calcination/sintering temperatures are different from that mentioned by the above authors. The use of these conventional methods could have impacted the obtained results, leading to the observed phases and structural variations in comparison to those in the referenced articles. Synthesis methods and sintering conditions are expected to influence the structural formations.

The XRD-refined parameters for all the studied compounds are listed in Table 1. It is observed that the cell volume of the LMMO compounds, *x* ≤ 0.15, is found to decrease systematically with the increase in Mg content, which is obvious as the Mg ion has a much smaller ionic radius than that of the La-ion. Further, the cell volume of the compounds with compositions *x* > 0.2 shows inconsistency with the evolution of a secondary phase. In previous studies on La-deficient manganites, an elevated concentration of Mn^4+^ within the compound is suggested to be responsible for a decrease in the unit cell volume.^42,43^ The research on A-site deficient and B-site deficient compounds within perovskite structures has been conducted extensively in the field of materials science.^20^ It is established that the creation of deficiencies in trivalent ions (A site) and divalent ions (B site) exerts opposing influences on the unit cell volume of stoichiometric compounds.^44^ The expansion of the unit cell volume resulting from A-site deficiency cannot be attributed to the quantity of Mn^4+^ ions; instead, it is linked to the ionic radius of the A-site, with a vacancy radius surpassing that of Mg^2+^ ions. This phenomenon occurs because the ionic radius of Mn^4+^ ions (0.53 Å) is smaller than that of Mn^3+^ ions (0.645 Å). The introduction of an A-site deficiency in manganites significantly influences the migration of the e_g_ electrons between Mn^3+^ and Mn^4+^ ions, similar to the changes in unit cell volume impacting the Mn–O bond lengths and Mn–O–Mn bond angles. As the quantity of Mg dopant at the A-site rises, a secondary phase gradually emerges. However, a further escalation in Mg concentration results in a heightened presence of the secondary phase. This behaviour is attributed to the smaller radius of Mg ions compared to La ions, which introduces an imbalance in the system by incorporating Mg ions.^45–48^

**Table 1:** XRD parameters obtained for LMMO sintered at 1250 °C

Sample parameters	*X* = 0.05	*X* = 0.10	*X* = 0.15	*X* = 0.20		*X* = 0.30		*X* = 0.33	
Crystal structure	Primary phase	Primary phase	Primary phase	Primary phase	Secondary phase	Primary phase	Secondary phase	Primary phase	Secondary phase
Space group	*R*3̄	*R*3̄	*R*3̄	*R*3̄	*I*4_1_/*amd*	*R*3̄	*I*4_1_/*amd*	*R*3̄	*I*4_1_/*amd*
Cell parameters (Å) *a*	5.5467 ± 0.00876	5.5272 ± 0.00743	5.5214 ± 0.0081	5.5257 ± 0.00672	5.7193 ± 0.00221	5.5278 ± 0.00534	5.7311 ± 0.001993	5.5303 ± 0.00537	5.7630 ± 0.001622
*b*	5.5467 ± 0.00876	5.5272 ± 0.00743	5.5214 ± 0.0081	5.5257 ± 0.00672	5.7193 ± 0.00221	5.5278 ± 0.00534	5.7311 ± 0.001993	5.5303 ± 0.00537	5.7630 ± 0.001622
*c*	13.4166 ± 0.03403	13.3444 ± 0.028	13.3204 ± 0.031	13.3343 ± 0.029	9.4717 ± 0.00159	13.3614 ± 0.00197	9.2733 ± 0.00210	13.3392 ± 0.002997	9.4610 ± 0.00105
Volume Å^3^	357.4690	353.0496	351.6794	352.5933	309.8215	353.3120	304.2203	353.5813	314.5851
*α*	90	90	90	90	90	90	90	90	90
*β*	90	90	90	90	90	90	90	90	90
*γ*	120	120	120	120	90	120	90	120	90
*R*_w_	55.7	43.9	52.8	46.2		50.2		50	
Goodness of fit	2.3	2.5	2.2	2.0		2.4		2.4	
*χ* ^2^	5.17	5.14	4.7	3.88		4.8		4.5	
% Phase fraction				96.84	3.16	94.62	5.38	90.87	9.13

A significant point to acknowledge is that the rhombohedral lattice can be described using a triple hexagonal cell, where its primitive cell is embedded inside a larger hexagonal unit cell, making it a supercell. Their translational symmetry can be described as with a unit cell *a* = *b* ≠ *c* and *γ* = 120°; however, this cell is not primitive. The hexagonal and rhombohedral crystal systems are closely related because the rhombohedral system is a subset of the trigonal crystal system, which is often described using the hexagonal setting. It contains three lattice points. Primitive description of the lattice is *a* = *b* = *c* with α = β = γ ≠ 90°. These space groups are symbolized with *R* as in *R*3̄*c*. The hexagonal system comprises 27 space groups. The trigonal system and its 25 space groups (143–167) belong either to the hexagonal (18 space groups) or the rhombohedral (7 space groups) Bravais lattice. All trigonal crystals with rhombohedral lattices (space groups 146, 148, 155, 160, 161, 166, and 167) can be represented as an equivalent hexagonal system; there is a choice of using a hexagonal or a rhombohedral representation.^49^

### 3.2 Field emission-scanning electron microscopy (FE-SEM)

**Fig. 3:**
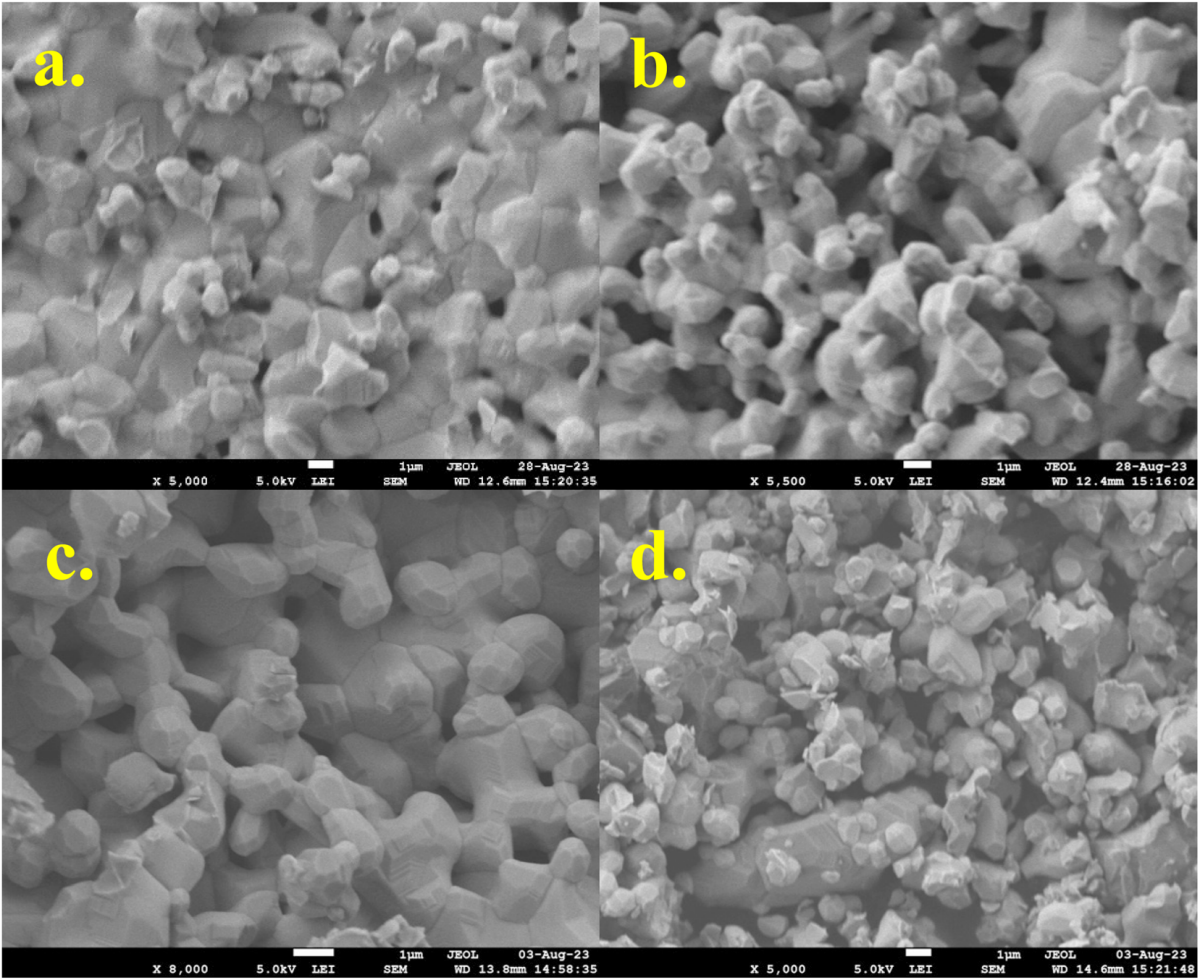
FE-SEM images of La_1−*x*_Mg_*x*_MnO_3_ (a) *x* = 0.05 (b) *x* = 0.1 (c) *x* = 0.2 (d) *x* = 0.3.

The FE-SEM analysis was performed to identify the surface morphology of the synthesized La_1−*x*_Mg_*x*_MnO_3_ (*x* = 0.05, 0.15, 0.2, and 0.3) samples, which is displayed in Fig. 3. The particles exhibited a polygonal morphology with an average grain size of 5 μm. The limited decrease in particle size can be attributed to the insufficient pressure applied during the grinding process, as the synthesis method employed was a solid-state method. We have also checked the compositional analysis and elemental mapping using Energy Dispersive X-ray spectroscopy (EDX) on the selected samples, which are shown in Fig. S1 as ESI.† The EDX spectra of the sample show that the compositions match well with the nominal compositions in the stoichiometric ratios. The elemental mappings of all the elements present in the samples are also shown in Fig. S2.† This mapping confirms the homogeneous elemental distribution in the samples without any significant aggregation. Further, no additional impurities were found. Overall, it appears that the morphological and compositional observations are consistent with the samples as expected.

### 3.3 Magnetic studies

**Fig. 4:**
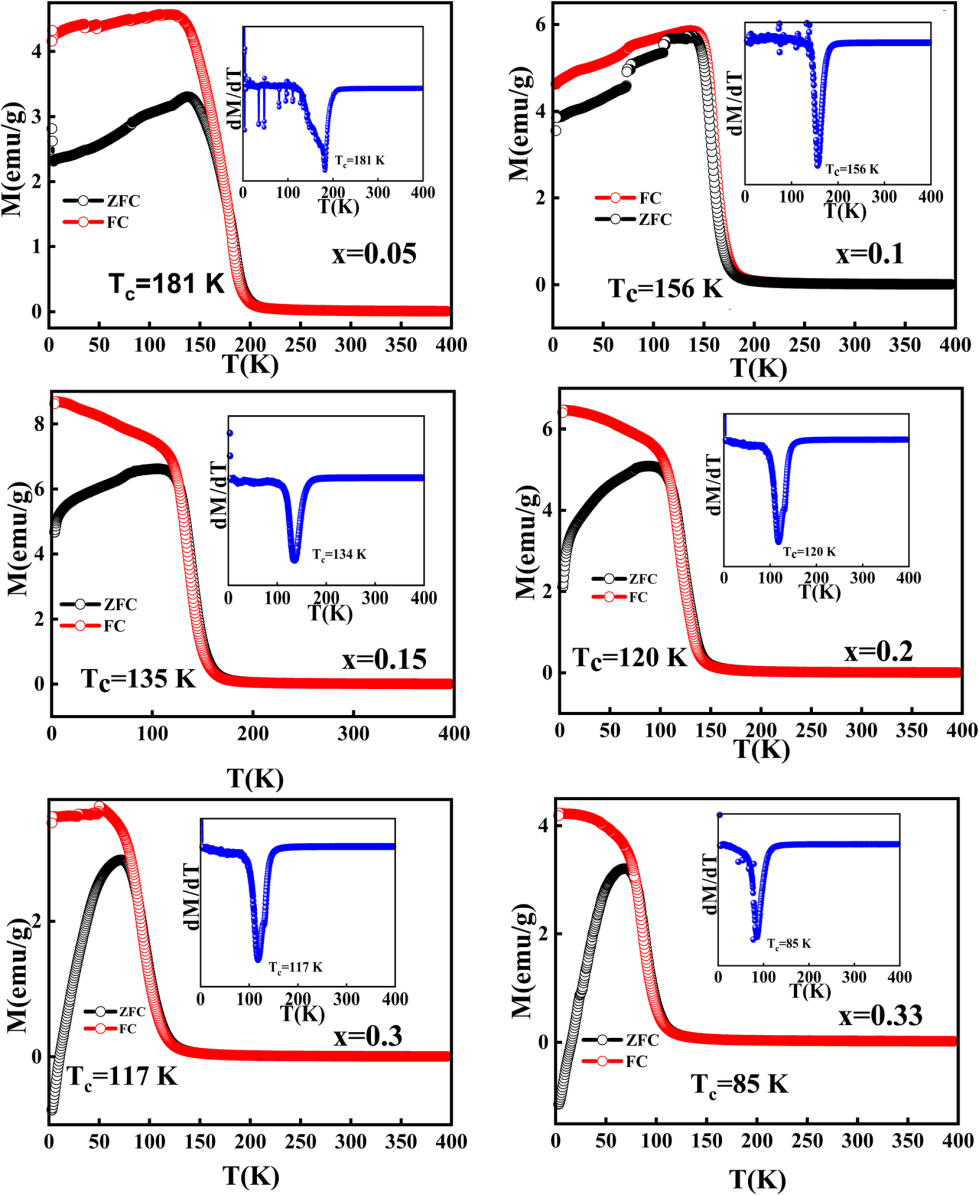
M–T plots of LMMO measured under ZFC and FC protocols. The insets show the derivative of the FC magnetization curves.

The temperature variations of the magnetisation curves, M(T), under the zero-field cooled (ZFC) and field-cooled (FC) protocols in the presence of a constant magnetic field, 100 Oe, were measured for all the studied compounds and depicted in Fig. 4. It is observed that the magnetic transition temperatures (*T*_c_) for all the samples are below 200 K. The *T*_c_ values are determined from the derivatives of the FC M(T) curves, which are shown in the insets of the respective compounds. The *T*_c_ values are found to be systematically decreasing with the increase in the Mg content (shown in Table 2). A clear divergence between the ZFC-FC M(T) curves is noticed for all the samples below *T*_c_, suggesting the co-existence of two different phases (as revealed from XRD studies) whose magnetic ordering may be different. As the Mg content in the La-site increases, the *T*_C_ is found to decrease owing to the crystallographic distortion induced by the different ionic radii between the La^3+^ and Mg^2+^ ions.^50^

**Table 2:** Composition-dependent magnetic and chemical properties of LMMO

Composition	*H*_C_ (Oe) (300 K)	*H*_C_ (Oe) (5 K)	*T*_C_ (K)
*X* = 0.05	—	—	181
*X* = 0.1	—	—	156
*X* = 0.15	—	—	135
*X* = 0.2	—	—	120
*X* = 0.3	76	350	117
*X* = 0.33	212	440	85

Further, a clear down-turn is noticed in the ZFC M(T) curves at very low temperatures for *x* = 0.15 compositions onwards and the same is witnessed to cross zero and enter into a negative magnetization for the compositions *x* > 0.2. The negative magnetisation obtained in the samples above *x* > 0.2 compositions indicates the existence of two different magnetic sublattices because of the evolution of the secondary phase of MgMn_2_O_4_, which is found to increase with the increase in Mg content in LMMO as revealed from the XRD results. MgMn_2_O_4_ is a spinel-structured compound and is reported to be ferrimagnetic in nature.^51^ The ferrimagnetic behaviour of MgMn_2_O_4_ suggests that the interactions between Mn ions and other ions in the crystal lattice produce a net magnetic moment. It is also hypothesized that some Mg^2+^ ions are still situated at the A sites, and antiferromagnetism may be caused by the B–B interactions, as A–B interactions, if present, are likely to be ferrimagnetic.^52,53^ In compositions below *x* < 0.2, where a pure phase of the perovskite manganite structure is formed, the net magnetic moment is due to the magnetic ordering of the primary phase alone, whereas for compositions *x* > 0.2, the net magnetic moment is due to the contribution of magnetic ordering from both primary and secondary phases. As it is known that the pure phase of LMMO is ferromagnetic, the magnetic ordering of the compounds below *x* < 0.2 compositions is understood to be due to the ferromagnetic contributions. However, in the compounds with the compositions *x* > 0.2, the contribution of ferrimagnetic ordering is enhanced; thus, the negative magnetisation is dominant in the ZFC M(T) curves. This is because the ferromagnetic and ferrimagnetic phases could be in the sublattices with opposite orientations. Ferrimagnetic materials often have opposing magnetic moments on different sublattices, which obviously affect the overall magnetic behaviour of the compounds. Therefore, the negative magnetisation in the ZFC M(T) curves is more prominently seen for the compounds with the increased fraction of the MgMn_2_O_4_ phase. Thus, the evolution of the MgMn_2_O_4_ spinel structure influences the magnetic properties, leading to a shift in the ZFC curve towards negative magnetization and a reduction in the overall magnetic behaviour of the material.

**Fig. 5:**
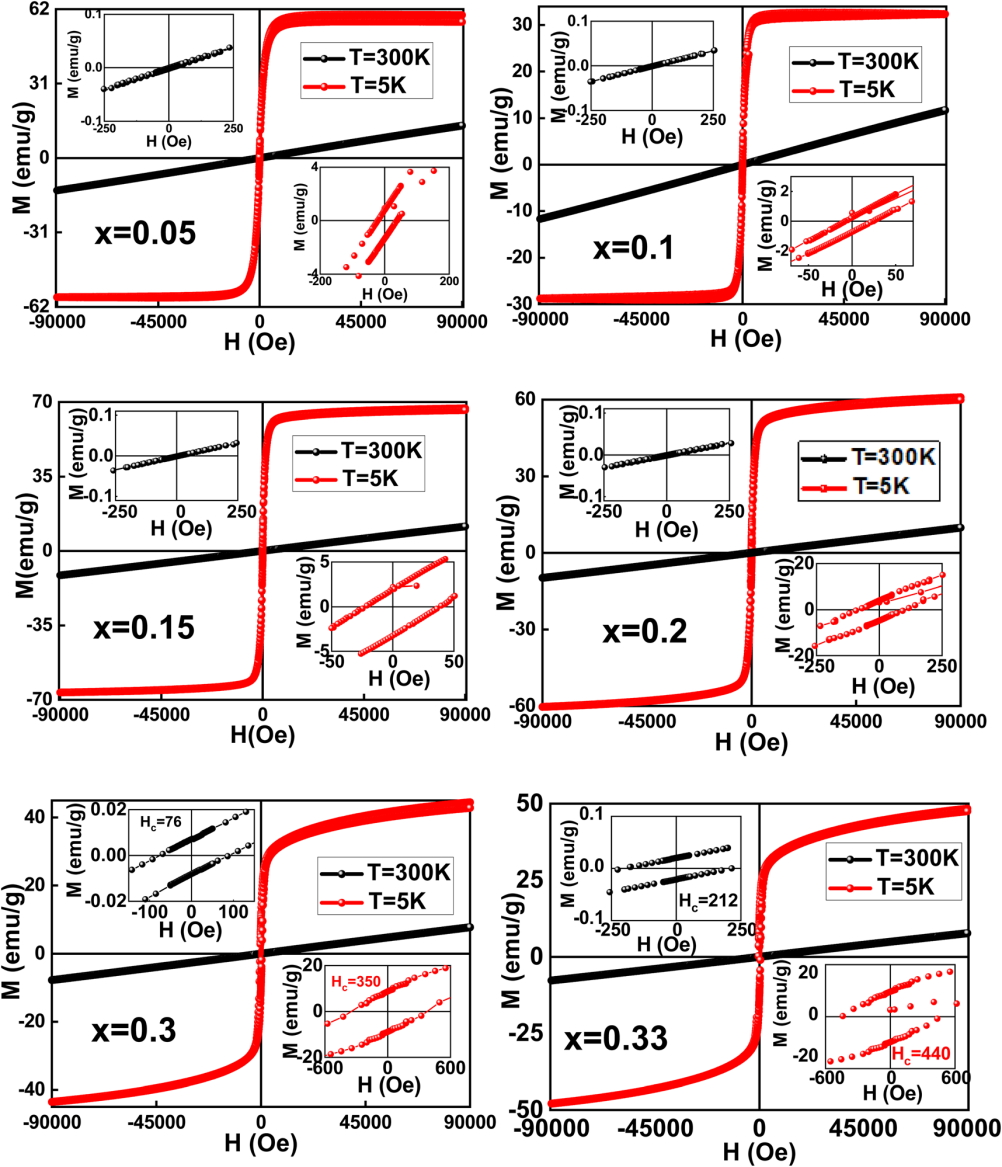
M–H plots of LMMO measured at 5 K and 300 K. The upper insets show a close-up view of the plots at 300 K, while the lower insets show a close-up view of the plots at 5 K.

Further, we have recorded the isothermal hysteresis loops at 5 K and 300 K, as shown in Fig. 5. It is observed that all the compounds show a straight curve at 300 K, confirming the paramagnetic nature. However, the compounds with *x* > 0.2 show a small hysteresis, which is absent in *x* < 0.2, as shown in the upper insets of Fig. 5 of the respective compositions. At lower temperatures, 5 K, all the hysteresis curves show a sharp increase in their magnetization with very low magnetic fields and then saturate with the further application of fields with finite coercivity values. Also, for *x* > 0.2 compositions, the samples are found to have a non-saturating tendency even with the application of higher magnetic fields of up to 90 000 Oe. This clearly indicates the presence of two magnetic components: one is FM, which quickly responds to the field and the other is ferrimagnetic, which responds very slowly and even gets saturated for the samples *x* < 0.2 and slowly increases for the *x* > 0.2 compounds without any saturation. Again, the coercivity values determined from the isothermal hysteresis loops measured at 5 K (shown in lower insets of Fig. 5 and Table 2) are found to increase sharply for *x* > 0.2 compounds, which can be attributed to the opposing interactions between the ferromagnetic and ferrimagnetic sublattices. This further corroborates with the ZFC-FC M(T) results. Thus, the magnetic results suggest that the net magnetic moment of the magnetisation obtained in all the studied samples is because of the magnetic ordering of the ferromagnetic characteristics for *x* < 0.2 compounds, whereas it is due to a co-existence of both ferromagnetic and ferrimagnetic ordering for *x* > 0.2 compounds.

**Fig. 6:**
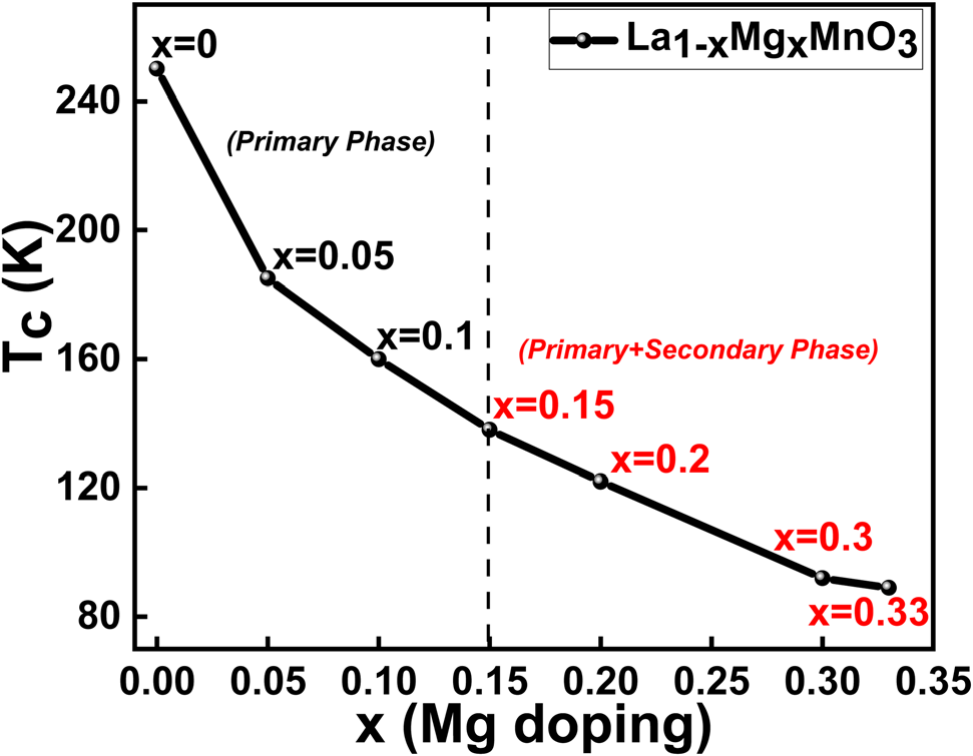
*T*_C_ *vs.* Mg doping concentration in LMMO.

The Mn^3+^ ions possess an electronic configuration of 3d^4^
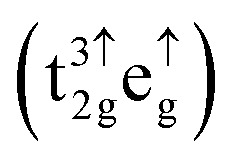
 with *S* = 2, while the Mn^4+^ ions have 3d^3^
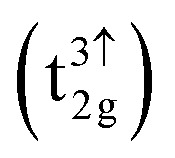
 electrons with *S* = 3/2.^54^ The spin-only magnetic moments are 4 μB for Mn^3+^ and 3 μB for Mn^4+^. Interestingly, Mn^3+^ is a Jahn–Teller ion that, because of its high Jahn–Teller action, causes octahedral distortion. Mn^4+^, on the other hand, is not a Jahn–Teller ion and does not deform the oxygen octahedra. The exchange coupling between the Mn^4+^ (3 d^3^) and Mn^3+^ (3 d^4^) ions is highly ferromagnetic in nature.^37^ In the present samples, as the Mn^4+^ ions increase with the increase in the Mg content, the overall ferromagnetic nature diminishes. Further, *T*_C_ decreases periodically with an increase in Mg doping and the reduction is explained by the Mg doping-induced increase in the unit cell volume (Fig. 6), confirmed by XRD analysis. This leads to the elongation of the Mn^3+^–O–Mn^4+^ chains and a weakening of the DE interaction. Additionally, Mg doping causes a tilt in the MnO_6_ octahedra, contributing to a reduction in the overlap between O-2p and Mn-3d orbitals, further contributing to the efficient reduction in *T*_C_. Thus, the magnetic properties of the LMMO are influenced by the presence of ferrimagnetic MgMn_2_O_4_ in the structure. Furthermore, isothermal hysteresis loops measured at 5 and 300 K revealed the presence of ferromagnetic and ferrimagnetic ordering in the presence of the MgMn_2_O_4_ phase for the higher Mg-content LMMO samples.

## 4. Conclusion

A series of Mg-substituted LMO manganite compounds (La_1−*x*_Mg_*x*_MnO_3_, with *x* = 0.05 to 0.33) were synthesised using solid-state synthesis and their structural, chemical, and magnetic properties were studied. A pure perovskite structure with a rhombohedral phase is formed for the compounds *x* < 0.15, but a secondary phase with a MgMn_2_O_4_ structure started evolving above *x* > 0.15. A systematic decrease in the ratio of Mn^3+^/Mn^4+^ ions with the increase in Mg content in LMO manganite confirms the structural stability and charge neutrality of the compounds. The detailed structural studies confirm the formation of the perovskite structure based on LMO for the lower Mg-content samples, while composite structures consisting of perovskite and a spinel structure based on MgMn_2_O_4_ are observed in the higher Mg-content samples. A systematic decrease in *T*_c_ with the increase in Mg-content and the net magnetic moment of the magnetisation obtained is due to the magnetic ordering of the ferromagnetic nature for the low Mg-content samples, whereas a co-existence of both ferromagnetic and ferrimagnetic ordering for higher Mg-content compounds. Therefore, the magnetic behaviour of the compounds is as per the structural formation of the compounds.

1. Mg-substituted LaMnO_3_ manganite compounds (La_1−*x*_Mg_*x*_MnO_3_, with *x* = 0.05 to 0.33) were synthesized using solid-state synthesis.

2. A pure perovskite structure with a rhombohedral phase is formed for compounds with *x* < 0.15, with the evolution of a secondary phase of the MgMn_2_O_4_ structure for *x* > 0.15.

3. A systematic decrease in the Mn^3+^/Mn^4+^ ratio with increasing Mg content confirms structural stability and charge neutrality.

The lower Mg-content samples exhibit a perovskite structure based on LaMnO_3_. With an increase in Mg content, the samples form a secondary phase combining the perovskite and a spinel structure of MgMn_2_O_4_.

A systematic decrease in the Curie temperature (*T*_C_) is observed with increasing Mg content. Lower Mg content samples display ferromagnetic ordering, contributing to higher net magnetic moments.

Higher Mg-content samples exhibit a co-existence of ferromagnetic and ferrimagnetic ordering.

Correlation of the structure and magnetism.

The magnetic behavior of the compounds aligns with their structural evolution, reflecting changes in magnetic ordering with Mg substitution.

## Data availability

The corresponding author (Dr M. Vasundhara) confirms that, upon request, the original data used in this manuscript will be provided.

## Author contributions

Parvathy Namboothiri: writing-original draft, methodology, investigation and conceptualization. Vishnumaya K. J.: investigation, methodology, and plotting. Phuong V. Pham: investigation and conceptualization. K. K. Supin: writing rough draft, conceptualization and partial analysis. M. Vasundhara: writing-review and editing, conceptualization, visualization, supervision, project administration, and funding acquisition.

## Conflicts of interest

The authors declare that they have no known competing financial interests or personal relationships that could have appeared to influence the work reported in this paper.

## Supplementary Material

RA-015-D4RA08238A-s001
