# Supplementary material for: Impact of Mg substitution in LaMnO3 manganites on their structural integrity and magnetic behavior
Source: RSC Adv. 2025 Mar 19;15(11):8561–71. doi: 10.1039/d4ra08238a (PMC11920968; doi:10.1039/d4ra08238a)
Supplement: RA-015-D4RA08238A-s001 [file RA-015-D4RA08238A-s001.pdf]

Supplementary Information (SI) for RSC Advances.
This journal is © The Royal Society of Chemistry 2025

Supplimentary Information

Impact of Mg substitution in $LaMnO_3$ manganites on structural integrity and magnetic behavior

Parvathy Namboothiri[1,2], Vishnumaya K J[1], Phuong V. Pham[3], K. K. Supin[1,2] and M. Vasundhara[1,2*]

[1]*Polymers and Functional Materials Department, CSIR-Indian Institute of Chemical Technology, Hyderabad-500007, India*

[2]*Academy of Scientific and Innovative Research (AcSIR), Ghaziabad-201002, India*

[3]*Department of Physics, National Sun Yat-sen University, Kaohsiung 80424, Taiwan*

[*]Corresponding authors: mvas@iict.res.in, vasu.mutta@gmail.com

1. Morphological Analysis

The surface morphology of the $La_{1-x}Mg_xMnO_3$ (x=0.05, 0.15, 0.2, and 0.3) samples synthesized was examined using FE-SEM analysis. Figure S1 depicts the Energy Dispersive X-ray (EDX) spectra of $La_{1-x}Mg_xMnO_3$ (x=0.05, 0.15, 0.2, and 0.3).

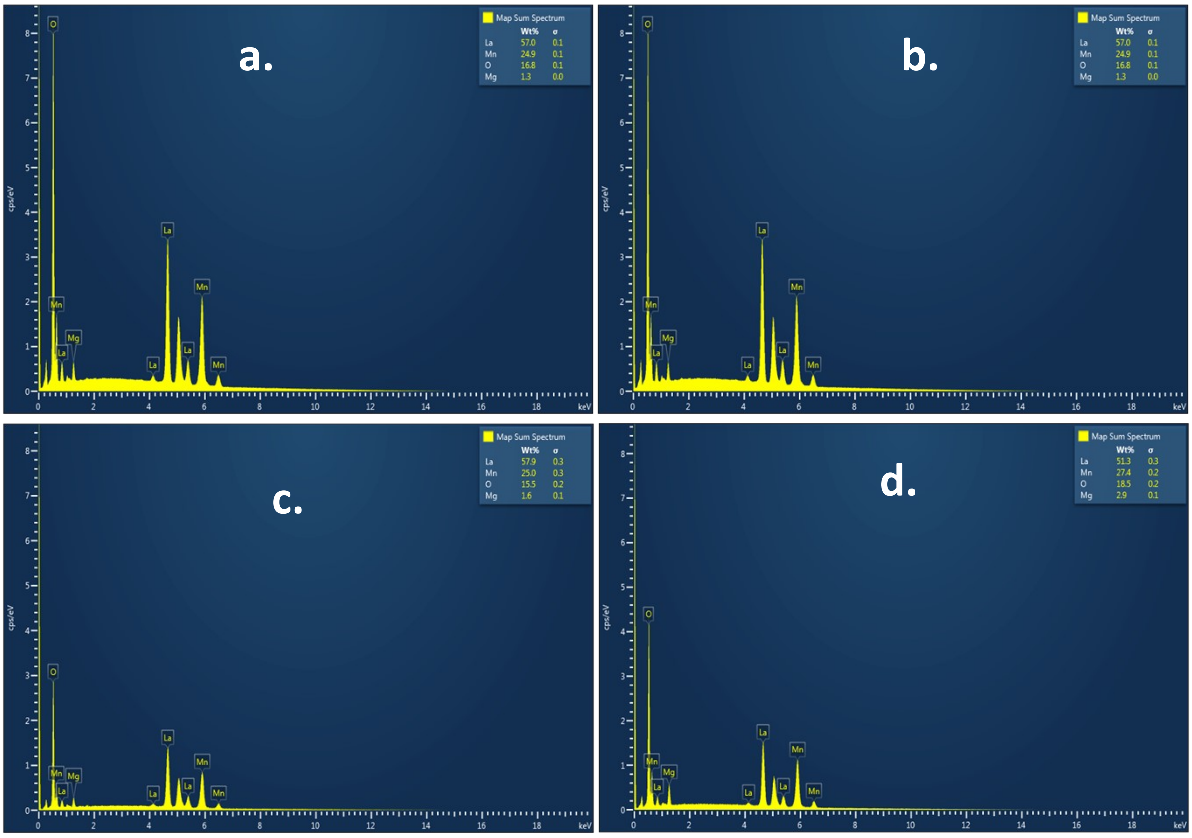

Figure S1. EDX spectra of $La_{1-x}Mg_xMnO_3$ (a)0.05 (b)0.15 (c)0.2 (d)0.3

EDX spectra exhibit peaks that enable both the identification and quantification of elements within the sample. Through these peaks,information regarding the elemental composition of

the sample can be derived, facilitating precise analysis and characterization. The high quality of the spectra indicates that the compositions possess a nearly pure phase chemical nature, with the desired components present in their respective compositions in stoichiometric ratios. No additional impurities were detected. These morphological and compositional observations align well with the predictions for the samples.

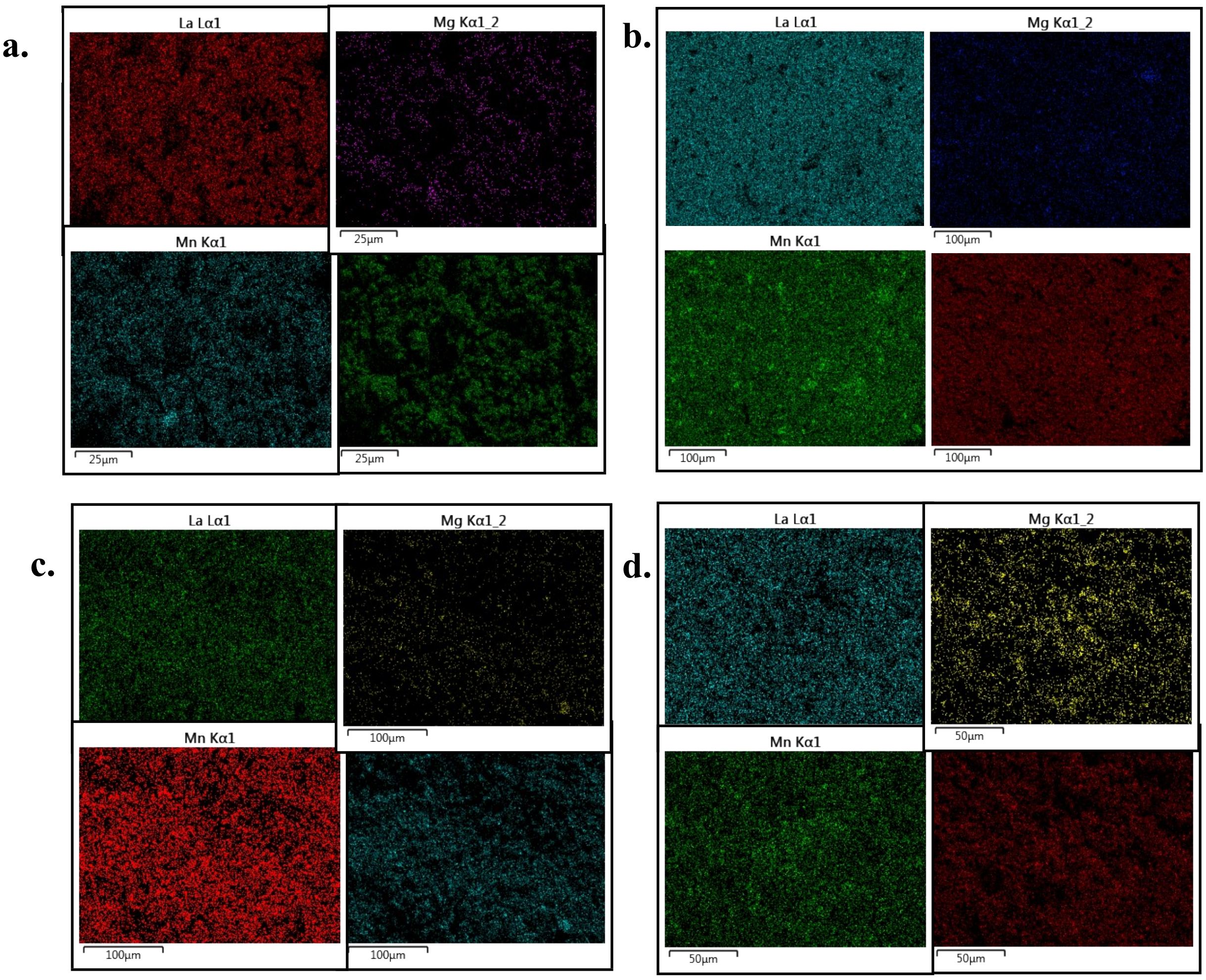

Figure S2. Elemental Mapping of $La_{1-x}Mg_xMnO_3$ (a)0.05 (b)0.15 (c)0.2 (d)0.3

Additionally, Figure S2 presents the elemental mapping for the $La_{1-x}Mg_xMnO_3$ (x=0.05, 0.15, 0.2, and 0.3) compound, confirming the uniform distribution of elements. Elemental analysis reaffirms the expected level of doping in the sample, maintaining stoichiometry. Moreover, the elemental mapping underscores the uniform distribution of component elements for each material.

2. Magnetic Studies

Below, we show the ZFC-FC plots of $LaMnO_3$ with Mg concentration 0.05, 0.1, 0.15. The T_c values are determined from the derivatives of the FC M(T) curves which are shown in the insets of the respective compounds. Further, the Tc values were also determined from the Curie-Wiess fit which is plotted in the right-axis of the same figure. The Tc values systematically decrease with the increase of Mg-content.

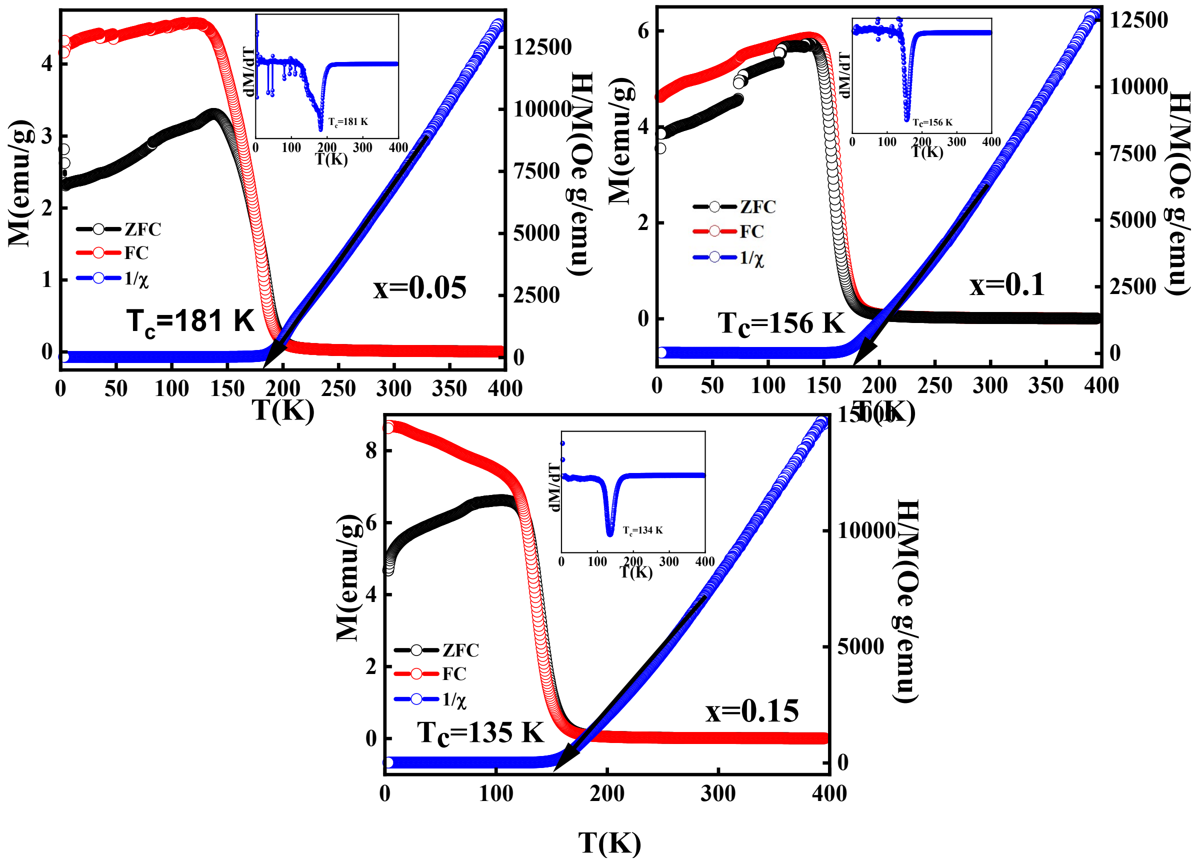

Figure S3: M-T plots of $La_{1-x}Mg_xMnO_3$ measured under ZFC and FC protocols. Insets of the figures show the derivative of the FC magnetization curves. Right-axis show the Curie-Weiss fit to the inverse susceptibility.
